# PEDOT-Coated PLA Fibers Electrospun from Solutions Incorporating Fe(III)Tosylate in Different Solvents by Vapor-Phase Polymerization for Neural Regeneration

**DOI:** 10.3390/polym15194004

**Published:** 2023-10-05

**Authors:** Laura S. Pires, Diogo S. Melo, João P. Borges, Célia R. Henriques

**Affiliations:** 1 Department of Materials Science, NOVA School of Science and Technology, NOVA University Lisbon, Campus de Caparica, 2829-516 Caparica, Portugal; ls.pires@campus.fct.unl.pt; 2 Department of Physics, NOVA School of Science and Technology, NOVA University Lisbon, Campus de Caparica, 2829-516 Caparica, Portugal; dso.melo@campus.fct.unl.pt; 3 i3N/CENIMAT, NOVA School of Science and Technology, NOVA University Lisbon, Campus de Caparica, 2829-516 Caparica, Portugal

**Keywords:** neural regeneration, electrospinning, Fe(III)Tosylate, PEDOT, vapor-phase polymerization, conductivity, SH-SY5Y neuronal growth

## Abstract

Therapeutic solutions for injuries in the peripheral nervous system are limited and not existing in the case of the central nervous system. The electrical stimulation of cells through a cell-supporting conductive scaffold may contribute to new therapeutic solutions for nerve regeneration. In this work, biocompatible Polylactic acid (PLA) fibrous scaffolds incorporating Fe(III)Tosylate (FeTos) were produced by electrospinning a mixture of PLA/FeTos solutions towards a rotating cylinder, inducing fiber alignment. Fibers were coated with the conductive polymer Poly(3,4 ethylenedioxythiophene) (PEDOT) formed by vapor-phase polymerization of EDOT at 70 °C for 2 h. Different solvents (ETH, DMF and THF) were used as FeTos solvents to investigate the impact on the scaffold’s conductivity. Scaffold conductivity was estimated to be as high as 1.50 × 10^−1^ S/cm when FeTos was dissolved in DMF. In vitro tests were performed to evaluate possible scaffold cytotoxicity, following ISO 10993-5, revealing no cytotoxic effects. Differentiation and growth of cells from the neural cell line SH-SY5Y seeded on the scaffolds were also assessed, with neuritic extensions observed in cells differentiated in neurons with retinoic acid. These extensions tended to follow the preferential alignment of the scaffold fibers.

## 1. Introduction

Whether because of neurodegenerative diseases or sudden injury to the Central Nervous System (CNS), or Peripheral Nervous System (PNS), millions worldwide suffer from impaired nerve function. Although nervous tissue has limited self-regeneration properties, there are some critical differences between PNS and CNS.

PNS regeneration heavily relies on the formation of fibrin cable between injured extremities. When a nerve is severed, Wallerian Degeneration (WD) takes place, clearing debris from the degraded axons at the distal stump. Meanwhile, Schwann Cells (SCs) proliferate from both nerve ends and aid Büngner band formation, which guides neurite outgrowth [1,2,3,4,5]. PNS can spontaneously regenerate small gaps up to 6 mm, but it usually ends with an incomplete regeneration, leaving the patient dealing with long-term consequences [6,7]. Jara et al. [6] presented an extensive text on the PNS regenerative response. When nerve suturing is impossible, grafting can be considered by using autografts, allografts, and xenografts by order of preference. However, these procedures do not come risk-free. There is a chance of donor nerve shortage, mismatch of nerve size, donor-site morbidity, undesired immune response and rejection [1,2,5,8,9,10]. Despite continuous effort, there is still no guarantee for full recovery [11]. This potential partial rehabilitation comes with various possible side effects, such as numbness, loss of sensory function or mobility, chronic pain, and permanent disability. These issues highlight the importance of tissue engineering solutions. Currently, there are several commercialized nerve guidance conduits (NGCs) used in the treatment of PNS injuries, such as NeuraGen, NeuraWrap, Neurolac and others. They provide alternative solutions to the current grafting techniques, being effective in preventing neuroma and scar formation and in reducing collateral neurite sprouting. However, these are still limited to the treatment of relatively small gaps (<4 cm) and are associated with poor functional recovery. Several strategies have been under research to improve NCGs [1], including the incorporation of guidance cues to SCs migration and axonal regrowth towards distal targets, stimuli to speed up axonal regrowth, and cell transplantation.

On the other hand, CNS regeneration is particularly changeling due to its inhibitory surroundings [1,5]. In the presence of Spinal Cord Injury (SCI), the first response is swelling of the spinal cord, which can disrupt blood flow and expand the already existing damage, leading to cell death, extreme inflammation, and the formation of scar tissue that restricts axonal outgrowth. With Traumatic Brain Injuries (TBI) and neurodegenerative diseases, the process is virtually the same, triggering an immune cascade reaction to the injury, preventing neuron proliferation. A detailed explanation of the CNS immune response system is given by Tian et al. [1] and Gordon et al. [3]. Despite the promising research that has been carried out to find strategies that promote CNS regeneration [10], current clinical treatments for CNS injuries are based on stabilization and there is no effective treatment.

Electrical Stimulation (ES) can induce a plethora of effects, from cell differentiation to improved wound healing [12,13,14], and so far, there have been reports of neurite outgrowth in vitro and nerve regeneration in vivo [10,12,15]. Yet, there is still no clear understanding of how electrical stimulation aids in neural regeneration [2,5,15,16,17,18]. Javeed et al. [2] presented a thorough literature review of the possible cellular pathways in which ES aids in axonal regeneration.

Synthetic polymer scaffolds have been reported as great support for neuronal regeneration [13,19,20]. By incorporating a conductive polymer in the scaffold, direct electrical stimulation of cells through the scaffold may promote and direct neurite outgrowth across the lesion site [13]. There are key requisites that tissue-engineered scaffolds must oblige to be a suitable alternative to traditional surgical methods. To minimize rejection and inflammatory responses, the scaffolds must have biocompatible and biodegradable properties. Moreover, the possibility to tailor them to mimic the native tissue ECM ensures their integration in the surrounding tissues while supporting cell adhesion, growth, and proliferation [5,10,13].

Prabhakaran et al. [20] produced a Poly(L-lactic acid)/Polyaniline (PLLA/PANi) scaffold to electrically stimulate Neural Stem Cells (NSCs) that were seeded onto the scaffolds. An electrical field of 100 mV/mm applied for a period of 60 min resulted in extended neurite outgrowth when compared with the control group of unstimulated NSCs. Huang et al. [21] produced Polypyrrole/Chitosan (PPy/Chitosan) scaffolds in which Schwann cells were seeded; cell adhesion and proliferation occurred with and without ES, showing that the scaffold itself is a suitable substrate. When SCs were under electrical stimulation, the measured levels of neurotrophins were much higher, suggesting that ES enhanced the expression and secretion of Nerve Growth Factor (NGF) and Brain-derived neurotrophic factor (BDNF). Babaie et al. [8] seeded rat Mesenchymal Stem Cells (MSCs) on a Poly(vinyl alcohol)/PEDOT:Polystyrene Sulfonate (PVA/PEDOT:PSS) scaffold produced by electrospinning. Cells adhered to the scaffold, and growth was observed. Their differentiation was studied under the application of an electric field, and when in the presence of differentiation factors, the expression of neuronal genes is improved. Pires et al. [18] found that human NSCs seeded on PEDOT:PSS scaffold crosslinked with 3-glycidoxypropyltrimethoxysilane (GOPS) successfully differentiated into neurons under electrical stimulation and that these neurons exhibited longer neurite length than differentiated neurons in the absence of stimulation. Zhao et al. [15] conducted an in vivo study on the impact of ES applied through implanted PPy/Silk Fibroin (PPy/SF) composite scaffolds in rat sciatic nerve. ES promoted axonal regeneration and restored nerve function with no inflammatory reaction in another in vivo study conducted by Song et al. [9], ES applied through PPy/Poly[(L-lactide)-co-(ε-caprolactone)] (PPy/PLCL) fibrous scaffolds bridging a gap in the sciatic nerve of a rat led to results similar to those obtained with the treatment based on autologous grafting. This alternative has the advantage of avoiding morbidity at the donor site associated with autografting. The group also analyzed in vitro PC12 cells seeded onto the scaffolds and verified that ES significantly increased neurite outgrowth.

Electrospinning is a cost-effective method that allows the production of nanofiber scaffolds that act as a support system for cell adhesion, growth, and proliferation. Scaffolds produced by electrospinning are widely accepted in tissue engineering due to the resemblance of their structure with that of the ECM, with a high surface area-to-volume ratio and high porosity (low mass-to-volume ratio). Taking advantage of electrostatic forces inside a charged jet of a polymeric solution, the electrospinning technique allows the production of scaffolds composed of fibers at micro and nanoscale [22], with adjustable porosity and mechanical properties [9,15]. In tissue engineering, it is of extreme importance that the scaffold possesses a structure similar to the ECM of the selected tissue and is composed of biocompatible materials, allowing for a more effective mechanical and biochemical support [5,22]. In neuronal tissue engineering, aligned fibers are preferred over random fibers, as they have proved to guide neurite growth, working as a topographical cue of the scaffold [1,8,23,24]. Results have been demonstrated in vitro and in vivo that aligned fibers facilitate Büngner band formation, Schwann cells (SCs) migration, and axon extension [15].

Poly(3,4-ethylenedioxythiophene) (PEDOT) is a Conducting Polymer (CP) formed by the polymerization of the monomer 3,4-ethylenedioxythiophene (EDOT). Among CPs, it has one of the highest electrical conductivity and stability in environmental conditions. PEDOT has shown promising properties of biocompatibility, particularly with neural and neuroblastoma cells [25,26]. Various methods can be used to obtain CPs, particularly PEDOT, the most common being chemical oxidation polymerization in solution, electrochemical polymerization and vapor-phase polymerization [27] (VPP). VPP is a technique in which the CP monomer in vapor form gets in contact with a substrate containing an oxidant. The polymerization reaction takes place in the monomer/oxidant interface. VPP has one major advantage when compared with the other two methods. VPP is solventless, which prevents any particle agglomeration and yields thin and homogenous films. VPP can also deposit CPs in non-conductive substrates [27,28].

In manufacturing conductive scaffolds for Tissue Engineering Applications, spincoating the substrate is often unsuitable as the essential scaffold porous structure may be buried. Bolin et al. [29] reported this situation for the chemical polymerization of PEDOT by spincoating a PET electrospun substrate with a solution containing both oxidant and monomer. Song et al. [9] reported that the structure of a Poly (l-lactic acid-co-ε-caprolactone) (PLCL) electrospun scaffold remained intact after being immersed in a monomer solution to which an oxidant solution was added under stirring, leading to PPy-coated PLCL fibers. Iandolo et al. [30] successfully coated a 3D-printed microporous medical-grade PCL scaffold with a conformal PEDOT layer via VPP, following scaffold immersion in an oxidant solution. A less frequently reported method to coat nanofibers with the oxidant solution is the electrospray of the oxidant on the scaffold [31]. Nonetheless, the procedure that appears to be the simplest is the electrospinning of a solution containing both the structural polymer and the oxidant, as described by Nair et al. [32] and Laforg et al. [33].

In literature, Iron(III) p-toluenesulfonate (FeTos) stock solutions at 40% *w*/*v* in butanol are predominantly used to incorporate the oxidant in the substrate prior to VPP. However, the use of other FeTos solvents may influence the properties of the produced PEDOT. Moreover, when electrospun fibers are produced containing the oxidant to be afterward used in EDOT VPP, an appropriate solvent system for both polymer and oxidant must be used and will impact the electrospinning process itself, as well as the resulting PEDOT coating of the fibers. In this work, we electrospun scaffolds from mixtures of PLA solutions in chloroform (CHL) or 2,2,2-Trifluoroethanol (TFE) and FeTos solutions in different solvents—Ethanol (ETH), N,N-Dimethylformamide (DMF) or Tetrahydrofuran (THF)—prior to EDOT VPP on the surface of the scaffolds fibers. To the best of our knowledge, it is the first time that the influence of the system of solvents in producing PLA-coated electrospun fibers by VPP following this approach has been investigated. Since our target is to develop a conductive scaffold to promote neural regeneration, the scaffolds were produced by electrospinning and collected in a rotatory cylinder in order to provide the fibers with a topographical cue for neurites extension. Fibers were analyzed regarding their morphology, the formation of PEDOT on their surface (chemical bonds), electrical conductivity and ability to support neuronal cells.

## 2. Materials and Methods

### 2.1. Solution Preparation and Scaffold Production

0.2 g of Poly(lactic acid) (PLA L175; Total Corbion PLA BV, Gorinchem, The Netherlands, MW = 209 kDa) was dissolved, under magnetic stirring overnight, either in chloroform (CHL; Carlo Erba Reagents, Emmendingen, Germany, >99%) or 2,2,2-Trifluoroethanol (TFE; TCI, Europe N.V., Zwijndrecht, Belgium, >99%) to obtain 2.5 g of PLA solution with a polymer concentration of 8% *w*/*w*. These solutions are labeled PLA_CHL and PLA_TFE, respectively, in Table 1.

100 mg of Iron(III) p-toluenesulfonate (FeTos; Santa Cruz Biotechnology, Inc, Heidelberg, Germany) were dissolved in ETH (LabChem, Zelienople, PA, USA), DMF (Carlo Erba Reagents, >99.9%), or THF (Carlo Erba Reagents, >99.9%). Mixtures of PLA and FeTos solutions, all with a polymer-to-oxidant ratio (PLA:FeTos) of 2:1 *w*/*w*, were optimized for electrospinning. It should be noted that a single solvent cannot be used to prepare PLA L175 solutions containing Fe(III)Tosylate since this oxidant is insoluble in the polymer’s solvents (CHL and TFE). Therefore, the electrospinning solution should be prepared using a binary solvent system, at least.

**Figure 1:**
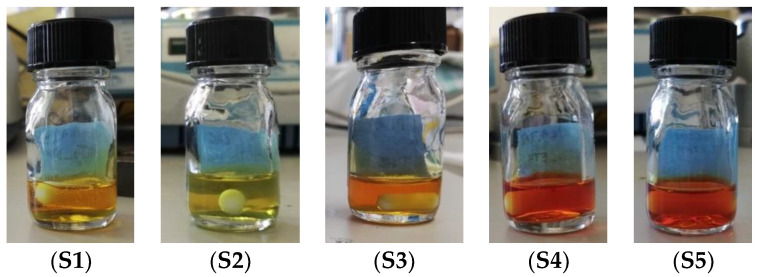
Electrospinning solutions. From left to right: (**S1**–**S5**).

The oxidant solution was added, drop by drop, under constant magnetic stirring, to the PLA solution 30 min before electrospinning. Five clear mixtures (S1–S5, shown in Figure 1), whose composition is presented in Table 1, were selected for electrospinning (scaffold precursor solutions). The scaffolds will be labeled after the solvents in their precursor solutions, as shown in Table 1.

For electrospinning, each Si (i = 1–5) solution was loaded on a 2 mL syringe attached with a 21 G stainless steel blunt needle (internal diameter of 0.508 mm). The syringe was placed in a syringe pump (SyringePump NE-300, New Era Pump Systems, Inc., Oyster Bay, NY, USA) to control the solution flow rate that was set at 0.5 mL/h. A high voltage power supply (T1CP300, iseg, Radeberg, Germany) was used to apply 18 kV to the needle tip relative to a homemade grounded rotatory cylindric collector (with a diameter of 8 cm) with a 25 cm distance in between. Electrospinning was performed under environmental conditions with temperature and relative humidity in the intervals 21 °C to 24 °C and 35% to 55%, respectively. Fibers were collected at two different collector rotation speeds: ~1750 rpm and ~3500 rpm.

### 2.2. Vapor-Phase Polymerization

Vapor-phase polymerization was carried out in a custom polymerization chamber with a maximum volume of 360 mL for 2 h at 70 °C. 4 mL of EDOT (Acros Organics, Geel, Belgium, 99%) was placed in an open container at the bottom of the chamber, while the scaffolds were placed at the top of the chamber. After polymerization, scaffolds were immersed for 30 min in ethanol 70% *v*/*v* to remove unreacted oxidants and monomers. After an overnight drying period, scaffolds were stored in a desiccator and tested after a time no longer than two weeks.

### 2.3. Fourier-Transform Infrared

To evaluate chemical bonds, the scaffolds were analyzed with an Attenuated Total Reflectance Fourier Transform Infrared Spectrometer (ATR-FTIR, Thermo Nicolet 6700, Waltham, MA, USA) in transmission mode. The spectra were acquired in a wavenumber range from 3500 cm^−1^ to 600 cm^−1^. Before each sample measurement, the background spectrum was acquired and afterward subtracted from that sample measurement to eliminate background noise.

### 2.4. Scanning Electron Microscopy (SEM)

SEM images were captured using an SEM-FIB Zeiss Auriga CrossBeam Workstation (Jena, Germany). Prior to image acquisition, all the samples were coated with a thin layer of iridium, ensuring a good electron outflow. Average fiber diameter and fiber angular distribution were evaluated from 100 and 50 measurements, respectively, performed in SEM images using the ImageJ 1.53 software (NIH, Bethesda, Rockville, MD, USA).

### 2.5. Electrical Characterization

To infer the electrical properties of the scaffolds, (*I*,*V*) data points were collected with a 2-point probe system, in-plane mode, using a Keithley 6487 Picoammeter/Voltage Source (Cleveland, OH, United States). Two parallel electrodes were painted with conductive silver paint (Pelco, Ted Pella, Fresno, CA, USA) in a 5 mm × 3 mm rectangular shape, separated by 2 mm. The (*I*,*V*) data were obtained for a range of applied voltages from −1 V to 1 V. Five measurements were performed on two replicas per material. Conductivity was estimated from Equation (1) [8], where l is the distance between the two electrodes, w is the width of the electrodes, d is the sample thickness, and R is the inverse of the slope of the linear fit to the *I*(*V*) plot.

(1) $$\sigma =\frac{l}{w.d.R}\;S/cm$$

### 2.6. In Vitro Evaluation of the Scaffolds

All the described culture procedures were performed inside a biological safety cabinet (Labculture Class II) for sterile work. Incubation of cells took place at 37 °C in a controlled atmosphere of 5% CO_2_ (MCO19AIC(UV)).

The scaffolds used for in vitro evaluation were tested after being submitted to the VPP process, as described in Section 2.2, and the samples were sterilized before testing. Sterilization was performed by immersing the samples in ethanol 70% *v*/*v* for 20 min and letting the samples dry inside the biological safety cabinet.

Hereinafter, the reference to DMEM means DMEM low glucose with stable glutamine and sodium pyruvate (DMEM Low, Sigma Aldrich, St. Louis, MI, USA), supplemented with 10 μg/mL gentamicin. The FBS supplementation of DMEM will be indicated as the *v*/*v* % FBS.

#### 2.6.1. Cytotoxicity Evaluation

Possible cytotoxic effects of the scaffolds were evaluated using the extract method according to the International Standard ISO 10993-5. To produce the extracts, sterilized pre-weighted samples of each scaffold were immersed in DMEM + 10% FBS, inside a sterilized tube, at a scaffold mass to medium volume of 15 mg/mL for an incubation period of 48 h.

VERO cells were seeded on the bottom of a 96-well plate (0.3 cm^2^ per well) at a density of $20\times 10^{3}$ cells/cm^2^ with DMEM + 10% FBS and incubated for 24 h. The culture medium was then replaced to proceed with the cytotoxicity test. Tested conditions included: the extracts DMEM + 10% FBS (for live-cell or negative control, C−) and DMEM + 10% FBS with 16% DMSO (dead-cell or positive control, C+). Cells were incubated in these conditions for an additional period of 48 h. 4 replicas of each condition were tested.

Cell viability was assayed using resazurin (Alfa Aesar, Haverill, MA, USA) as a cell viability indicator. The extracts were replaced by a (1:1) *v*/*v* mixture of a resazurin solution (0.04 g/mL in PBS) and complete DMEM. This mixture was also dispensed in empty wells to be used as a reference. After an incubation period of 3 h, absorbance was read at 570 nm and 600 nm (Biotek ELX 8000UV, Winooski, Vt, United States). The average values of 570 nm are subtracted from the average values of 600 nm to obtain the corrected average absorbance readings and to evaluate the relative viability from the ratio of these absorbances measured for the tested conditions and the negative control.

#### 2.6.2. SH-SY5Y Growth on Scaffolds

Samples of the electrospun scaffold CHL_DMF washed and not subjected to VPP, i.e., comprising PLA fibers not coated with PEDOT (control-PLA), and samples of all the scaffolds subjected to VPP samples were used in neuronal cell cultures. An 8% *w*/*w* solution of PLA in CHL was used to fix circular samples of the scaffolds to coverslips (12 mm in diameter). For that, a thin strip of the solution was carefully applied on the sample border with a syringe equipped with a blunt needle while keeping the sample in place until solvent evaporation. The samples fixed to coverslips were sterilized with ethanol 70% *v*/*v*, following the procedure described in Section 2.6, and placed in custom-made Teflon inserts for a 24-well plate, with a culture area of 0.5 cm^2^ and using a working volume of 200 μL, to evaluate how neuronal cells grow on the scaffolds.

Cells from the neural cell line SH-SY5Y, after neuronal differentiation with Retinoic Acid (RA), were used as model neurons. SH-SY5Y cells were first seeded in DMEM + 10% FBS (proliferation medium) at a density of $8\times 10^{3}$ cells/cm^2^ on the scaffolds, as well as on coverslips and wells bottom that were used as controls, and incubated for 24 h. Subsequently, cells were exposed to a differentiation medium, and the culture proceeded for 12 days, with the medium being changed every 48 h. A differentiation medium was prepared with DMEM + 3% FBS where RA dissolved in DMSO at a concentration of 2 mM was diluted by a factor of 200 to achieve a 10 μM concentration in the culture medium (and a non-toxic DMSO concentration of 0.5%).

#### 2.6.3. Cell Imaging

The morphology of the cells in the controls over the culturing time was assessed using an inverted microscope (Eclipse Ti-S, Nikon, Tokyo, Japan) equipped with a camera to assess bright field images.

At day 13 after seeding, cells growing on the scaffolds samples and cover slips (control-coverslip) were fixed with 3.7% *v*/*v* paraformaldehyde in PBS, permeabilized with a solution of 0.5% *v*/*v* Triton X-100 in PBS and stained to observe the cytoskeleton, with Acti-stain 555 Phalloidin (Cytoskeleton, Inc.100 nM in PBS, Denver, CO, USA), and to observe the nucleus, with DAPI (Molecular Probes, 300 nM in PBS). All samples were mounted on glass coverslips with antifade mounting medium (Booster) and imaged with the microscope Nikon Ti-S equipped with an epi-fluorescence module.

## 3. Results and Discussion

### 3.1. Scaffold Production

Before establishing the composition of the solutions to be electrospun, we found that mixtures of the solvents used in S1–S5 allow the dissolution and electrospinning of PLA within certain ratios. For instance, the mass ratio of 4.6:1 of CHL:DMF is adequate for electrospinning a PLA solution, but a decrease in that ratio leads to polymer gelation, which can be attributed to the strongly swollen PLA in the DMF [34]. Since FeTos is insoluble in the polymer’s solvents (CHL and TFE), we had to carefully adjust the contents of the PLA solution and FeTos solution in order to obtain clear solutions that could be electrospun. We set a (PLA:FeTos) ratio at 2:1 *w*/*w* for the electrospinning solution and arrived at the five solutions (S1-S5) that can be seen in Figure 1 with composition presented in Table 1 and the preparation mode described in Section 2.1.

Fibrous scaffolds were successfully electrospun from solutions S1–S5. 

**Figure 2:**
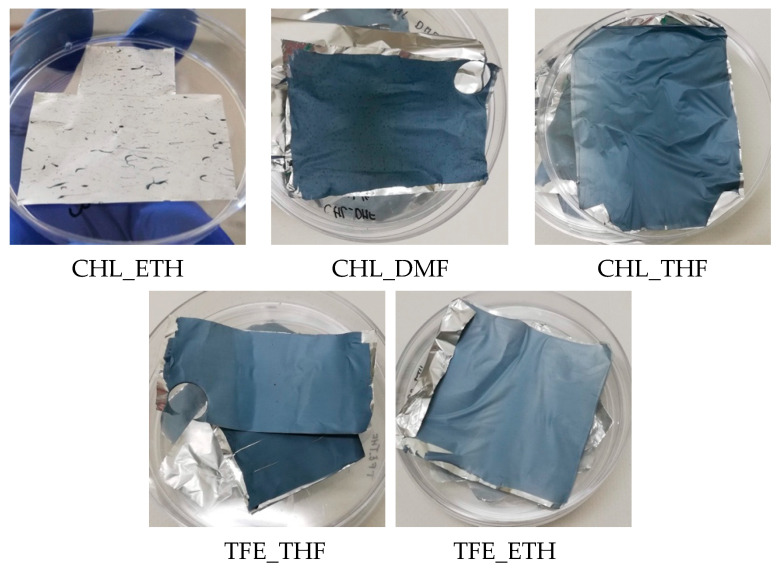
Post-polymerization scaffolds were submitted to VPP for 2 h at 70 °C.

Except for the CHL_ETH scaffold, after 2 h VPP all the other scaffolds present some degree of EDOT polymerization all over the scaffolds, observed by the color change from yellowish white to blue hue (characteristic of PEDOT), as displayed in Figure 2. The CHL_ETH only exhibits blue drops dispersed throughout the sample, which may be due to an inadequate dispersion of the oxidant in the electrospinning solution, and it was discarded.

The use of different systems of solvents originated dissimilar shades of blue, particularly when comparing CHL_DMF (dark blue) with TFE_ETH (light blue). Dark blue PEDOT is associated with the conductive polymer (doped state), whereas light blue PEDOT is associated with its oxidized non-conducting state. Differences in the blue tone of PEDOT can also occur due to the conductive polymer thickness; the thicker the layer, the darker it gets [29]. If there were no differences in the morphology of the scaffolds (same fiber diameter, thickness and uniformity of PEDOT layer), a darker scaffold should mean a more conducive one resulting either from longer polymer chains and/or higher doping level. These factors depend on the oxidant/dopant species in the solution.

It is noticeable that the FeTos solutions have distinct macroscopic characteristic colors: FeTos solution in DMF presents a much lighter (yellowish) color than that in THF, and the difference is even bigger when ETH is used (yellow-reddish). The discrepancy in the color of the DMF-containing solution to the others is still evident after mixing the oxidant solution with the polymer solution (see Figure 1), suggesting the presence of different iron species/complexes with different redox properties. An identical color discrepancy was observed by Hojati-Talemi et al. [35], who studied the influence of DMF in the VPP of EDOT on the surface of substrates produced by spin coating of solutions containing FeTos: when butanol/ethanol was replaced by DMF in the solution, the color turned from a dark orange/brownish to yellow. The use of polar aprotic solvents (such as DMF) was linked in this study to changes in the coordinating shell of iron, leading to changes in the redox potentials [35,36]. These changes control the number of accessible polymerization nucleation sites that directly affect the polymerization rate, PEDOT grain size, and consequently the properties of the conductive polymer: a reduced number of nucleation sites leads to a slower polymerization rate reaction, whereas a thinner PEDOT coating is obtained with larger grains. In the work of Hojati-Talemi et al. [35], the substitution of butanol by DMF in the solvent resulted in larger PEDOT grains and a thinner PEDOT layer, whether water (a polar protic solvent) leads to opposite results.

A widely accepted mechanism for PEDOT polymerization in a VPP chamber using FeTos as an oxidant consists of a 2-step reaction occurring in a loop [37]. In the first step, the EDOT in gaseous form contacts the FeTos, the monomer is oxidized, and the iron is reduced. In the second step, the oxidized EDOT monomers form dimers which are subsequently deprotonated by water molecules. The polymer chains are formed by a continuous loop of these two steps: the remaining iron in FeTos molecules continues to oxidize the chain while the tosylate molecules act as counter-anions. This mechanism requires the presence of water that, in some works reported in the literature, is added to the polymerization chamber [38]. Even when no water is added neither to the oxidizing solution nor in the polymerization chamber, as in the case of this work, Mueller et al. [37] found that the presence of small amounts of water in the atmosphere, complexed with FeTos or dissolved within the solvents, provides the necessary water for the polymerization process. In our work, the VPP chamber was closed at the ambient conditions (relative humidity between 35–55% and temperature between 21–24 °C) that determined the availability of water molecules in the atmosphere inside the VPP chamber.

From the above analysis, we can conclude that different solvent systems will influence the iron species’ redox state and, consequently, the properties of PEDOT. However, the exact analysis of those influences and comparison for the solutions under investigation is hampered by the possibility of the development of copious iron complexes in our multicomponent systems involving water and four categories of solvents—non-polar aprotic solvent (CHL), non-polar protic (TFE), polar aprotic (DMF and THF) and polar protic (ETH)—and many possibilities of iron complexes being formed. However, the simple observation of different solutions’ colors confirms differences in the chemistry of iron present in the process.

### 3.2. Scaffolds Morphology

**Figure 3:**
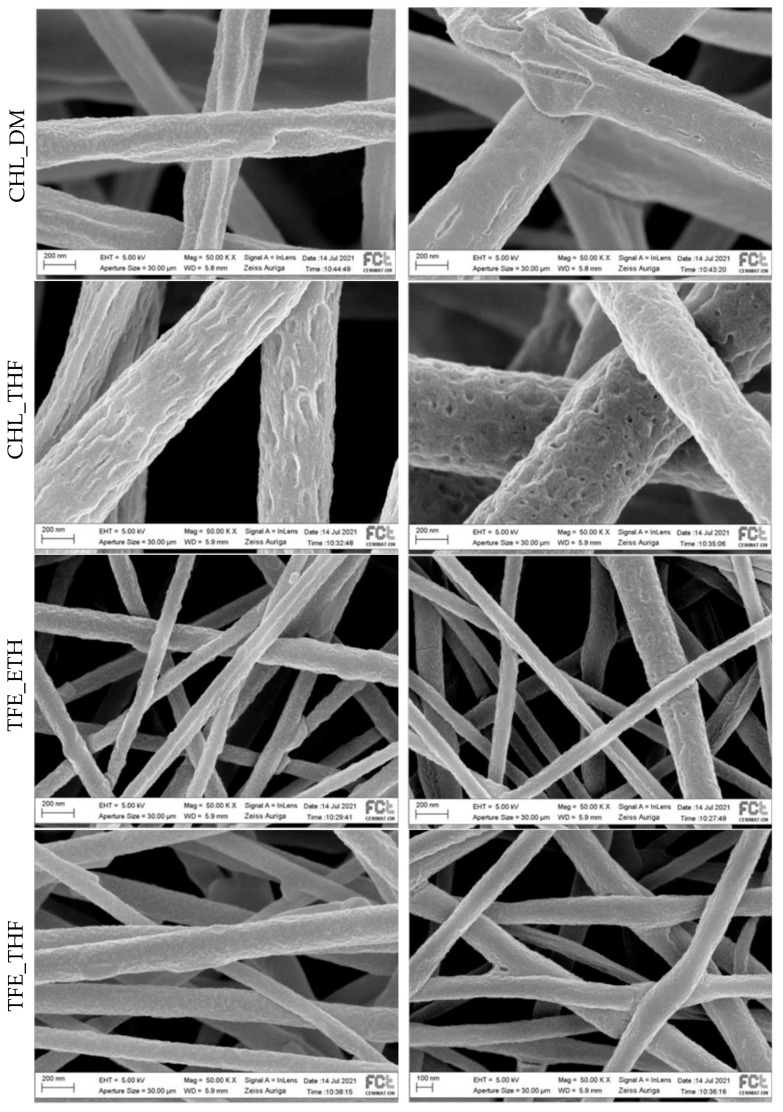
SEM images of pre-polymerization (**left**) and post-polymerization (**right**) of the fibers from the different scaffolds collected on the rotatory cylindric collector at ~1750 rpm, from top to bottom: CHL_DMF, CHL_THF, TFE_ETH and TFE_THF.

SEM images of the scaffolds collected on the rotatory cylindric collector at ~1750 rpm pre and post-polymerization are presented in Figure 3.

Fibers electrospun from solutions containing PLA_CHL are thicker than those electrospun from PLA_TFE-containing solutions, which is expectable due to the very low conductivity and high volatility of CHL. Considering the work of Fryczkowski et al. [39], thinner fibers are expected to be obtained when solutions with a higher conductivity are used since conductivity impacts the fiber stretching as a consequence of charge repulsion inside the jet itself.

The average fiber diameter and standard deviation obtained for the pre-polymerization and post-polymerization fibers were, respectively, (410 ± 199) nm and (463 ± 71) nm for CHL_DMF, (481 ± 195) nm and (596 ± 122) nm for CHL_THF, (116 ± 45) and (94 ± 17) nm for TFE_ETH, and (139 ± 47) and (156 ± 32) for TFE_THF. Despite the high values of the standard deviation, the mean fiber diameter tends to increase after VPP. The only exception is TFE_ETH, which may be a greater relative uncertainty in the measurements of these smaller values. The trend towards an increase in the average fiber diameter, together with the observed change in the surface fiber morphology, denotes the formation of PEDOT by VPP around the fibers, also revealed by the scaffold’s color.

Oxidant availability is the limiting factor for VPP [40]. Assuming a uniform distribution in volume of the oxidant, and no other variables, since the proportion PLA: FeTos is the same for all scaffolds, the surface density of the oxidant should be independent of the fiber diameter. In this scenario, we would expect a thickness of the PEDOT-coating identical for all fiber diameters. However, the exact way how the oxidant nucleation centers are distributed in the polymer fibers is not known—this distribution may be affected by multiple interactions and by the electrospinning process. The range of penetration of the EDOT in the fibrous structure may vary from scaffold to scaffold, as well as the thickness of the scaffold. The conducting properties of the scaffolds should be a result of several uncontrolled variables.

The uniformity of PEDOT-coating may also contribute to ensuring better electrical continuity and, consequently, to better conductance. In this respect, the surface of the PEDOT-coating the CLH_DMF fibers seems to be the most uniform, and that of the CLH_THF is the less one. Beyond the irregular surface morphology of the uncoated fibers, resulting, for instance, from different evaporation rates of phase-separated solvents (such as CHL and water), the high water-affinity of FeTos may also affect the uniformity of the fibers PEDOT coating. This water contributes to FeTos crystallization, and since crystals do not participate in the polymerization reaction, the PEDOT formed in substrates with crystals presents a much more defective topography, exhibiting holes, cracks and an irregular surface [41].

**Figure 4:**
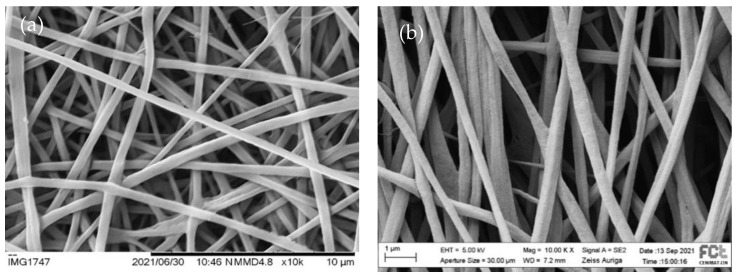
SEM images of the pre-polymerized CHL_DMF fibers collected on the rotatory cylindric collector (**a**) at ~1750 rpm (randomly aligned fibers) and (**b**) at ~3500 rpm (fibers with some degree of alignment).

Because aligned fibers provide an important guidance cue for neuronal regeneration, we used a rotatory cylindric collector to induce fiber alignment. Two rotational speeds were used (~1750 rpm and ~3500 rpm). Figure 4 illustrates the impact of the increase of the collector speed on fiber orientation by showing representative pictures of the fiber electrospun from the same solution under the same set of electrospinning parameters but with the rotation collector speed.

A higher fiber alignment is noticeable for the higher rotation speed, although there is still some dispersion in the angular distribution. Similar results were found for all the other scaffolds (images not depicted) and can also be found in the literature. Voniatis et al. [42] observed an increase in the degree of alignment of poly(vinyl alcohol) fibers produced by electrospinning, with an increase in the rotation speed of a cylindrical collector up to 6000 rpm. From the angular distribution of the fibers in the scaffolds produced in this work at the collector rotation speed of 3500 rpm, we estimated the percentage of fibers oriented within a range of 60 degrees. The results are 94% for CHL_DMF, 76% for CHL_THF, 74% for TFE_THF and 87% for TFE_ETH. Among the electrospinning solution parameters (conductivity, viscosity, surface tension and solvent volatility), conductivity has a major impact on the orientation of fibers [43] electrospun from the different solutions under the same process parameters. The higher degree of alignment for the CHL_DMF fibers may be due to the low conductivity of CHL (<10^−10^ S/cm [44]) and DMF (6 × 10^−8^ S/cm [45])—a high solution conductivity leads to an increased stretch of the polymer jet and consequently to a long length yarn. Therefore, a high rotational speed is needed to wrap the yarn in parallel circles around the periphery of the cylinder’s length. It should be noted that the presence of the oxidant in the solutions contributes significantly to higher conductivity and to a more difficult fiber alignment.

### 3.3. Fourier Transform Infrared Spectroscopy

**Figure 5:**
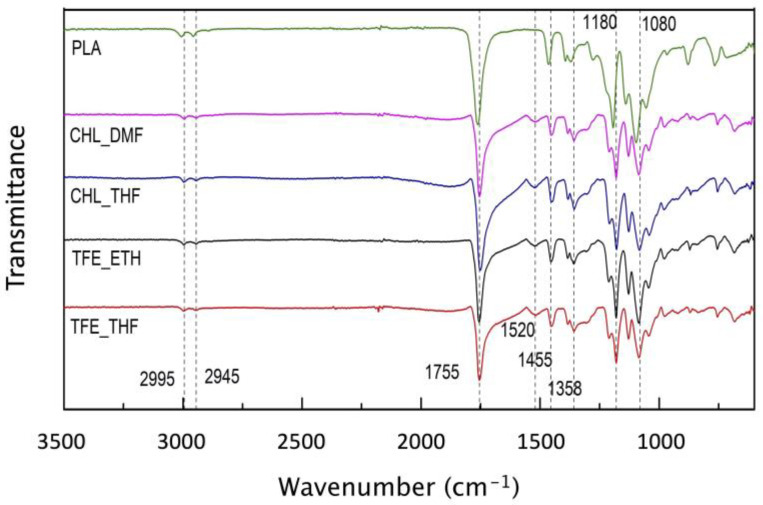
FTIR spectra of Pristine PLA (green), CHL_DMF (pink), CHL_THF (blue), TFE_ETH (black), and TFE_THF (red) scaffolds from 3500 cm^−1^ to 600 cm^−1^.

Figure 5 shows the FTIR spectra of pristine PLA and the post-polymerized scaffolds.

Characteristic peaks (marked with a dotted line across the graph) for pristine PLA are at 2995 cm^−1^ and 2945 cm^−1^, which represent the stretching frequencies of asymmetric and symmetric -CH_3_; 1755 cm^−1^ for the C=O stretch, and 1080 cm^−1^ for the C-O stretch. The peaks associated with the bending of the asymmetric and symmetric -CH_3_ are located at 1455 cm^−1^ and 1358 cm^−1^ [46,47]. The development of a peak at 1520 cm^−1^, which is not present in the PLA spectrum, is a result of the conjugated C=C system of the PEDOT [38,48].

The lack of a peak at around 3110 to 3116 cm^−1^ evidences the polymerization of the EDOT monomer since the 2,5-hydrogens are removed from the thiophene ring during the polymerization reaction. The work of Fabretto et al. [38] supports this evidence as the peak observed at 3110 cm^−1^ in their samples was attributed to the presence of dimers, trimers and oligomers rather than large chains. The development of a peak at 1520 cm^−1^, as previously stated, is a result of the conjugated C=C system of PEDOT [38,48]. It is common in VPP processes to have an unwanted side reaction due to the innate acidity of the FeTos, that in turn promotes cleavage of the dioxane ring due to the formation of C=O groups. Instead of conducting PEDOT, a non-conjugated and non-conductive polymer is obtained that can be identified by the formation of a peak from 1700 cm^−1^ to 1730 cm^−1^ [38,48,49,50,51]. A subtle shoulder peak is seen covering this region. Since the broad absorption peak characteristic of PLA at 1755 cm^−1^ can overlap this region, the observed shoulder peek raises the possibility of the occurrence of the unwanted process. A strategy to bypass this acidic-driven polymerization is the use of a base inhibitor with high vapor pressure, such as pyridine, to make the pH decrease allowing conducting PEDOT formation before the uncontrolled polymerization takes place [49].

### 3.4. Electrical Characterization

The values of scaffolds conductivity estimated following Equation (1) are presented in Table 2.

VPP PEDOT, provides scaffolds with some conductivity (PLA scaffolds are not conductive, as we confirmed). All the conductivity values obtained are in accordance with those reported for scaffolds used for cell stimulation and neural regeneration in vitro and in vivo [8,9,15,18,20,29]. Although the method followed in this work to estimate conductivity (see Section 2.5) of the electrospun scaffolds has been used in other works for the same purpose [8], both the experimental method and the interpretation of the calculated results poses challenges. Concerning the method, it is challenging to manipulate the membranes and restrict the painted region to a given regular electrode geometry and to avoid current conduction through painting infiltrated in the fibrous structure. For the interpretation of the results, we should have in mind the non-uniform structure of the volume of the scaffold (composed of fibers and holes) and the non-uniform composition of the fibers and scaffolds (a PEDOT coating wrapping PLA fiber mainly at the surface of the scaffolds). Such a structure and composition limits the approximation underlying Equation (1) which is based on Ohm’s law, to a uniform block of a conducting material. Although the model is not the best, it somehow accounts for the normalization of the sample sizes to compare the average electrical behavior of the material. To analyze the impact of different electrical stimulation conditions imposed on cells through a conductive scaffold device, more important than defining a property to characterize the material of the device is to measure the electrical currents involved in the cell stimulation under the experimental conditions (with the polarized scaffold in the in vitro culture conditions). Currents passing through the tested scaffolds go up to about 1 mA.

Despite all the factors discussed above limiting the clarification of the reasons for the scaffold’s conductivity found, some correlations may be hypothesized. The CHL_DMF scaffold is the one with fibers exhibiting a more uniform PEDOT-coating that, together with its dark-blue color, suggests its highest conductivity. The differences in the CHL_DMF and CHL_THF solution colors may indicate significant differences in their conducting properties. The low conductivity of CHL_THF may be due to the non-uniform PEDOT-coating formed at the fiber’s surface. The light blue color of TFE_ETH and TFE_THF indicates a lower conductivity. Differences between TFE_ETH and TFE-THF may be justified by the different nature of the solvents of the FeTos solution—a polar protic solvent in the case of TFE_ETH and a polar aprotic solvent in the case of TFE_THF.

### 3.5. In Vitro Evaluation

#### 3.5.1. Cytotoxicity Assay

**Figure 6:**
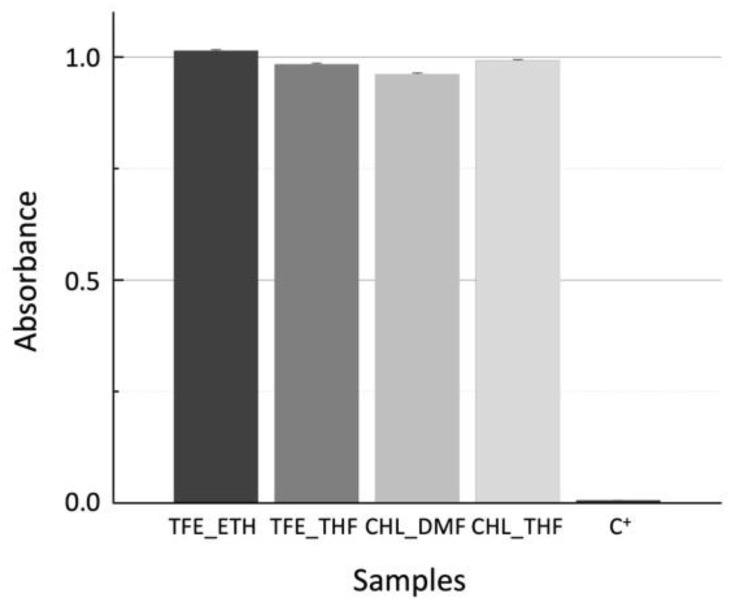
Corrected average absorbance readings, normalized to C^−^, for Vero cells incubated 48 h with scaffold extracts obtained by placing samples in culture medium at a ratio of 15 mg/mL.

The results of the colorimetric resazurin assay performed with Vero cells incubated in contact with the scaffold extracts produced at a scaffold mass to medium volume of 15 mg/mL are shown in Figure 6.

Resazurin is a blue dye that can be reduced by metabolically active cells to resorufin, a pink compound. The corrected absorbance is a reduction index that can be correlated to cell viability and to cytotoxicity effects. The results are normalized to the C− control (viable cells seeded at the bottom of plate wells). No cytotoxic effects were observed since all the samples have cell viability values higher than 95%. The validity of the test is confirmed by the extremely low cell viability, 0.6%, of the dead-cell control (C+).

#### 3.5.2. SH-SY5Y Growth on Materials

The cell line SH-SY5Y is derived from human neuroblastoma and is often used as a neuronal model. It is composed of a mixed population of floating N-type neuroblast-like cells and a smaller percentage of adherent S-type epithelial-like cells. Undifferentiated SH-SY5Y cells present an epithelial-like phenotype, grow in clusters, have high proliferation rates and present short processes pointing outwards. These cells can differentiate from a neuroblastic state into mature human neurons. During the differentiation process, neuritic extensions are formed, proliferation rates decrease dramatically, and a polarized shape can occur. Even though several studies are conducted using undifferentiated SH-SY5Y cells to obtain the most accurate comparison with in vivo models, it is important to use these cells in their matured neuron state [52,53,54,55,56,57,58], as in the work of Bolin et al. [29], we used SH-SY5Y cells as neuron models in this work for testing cell–material interaction.

**Figure 7:**
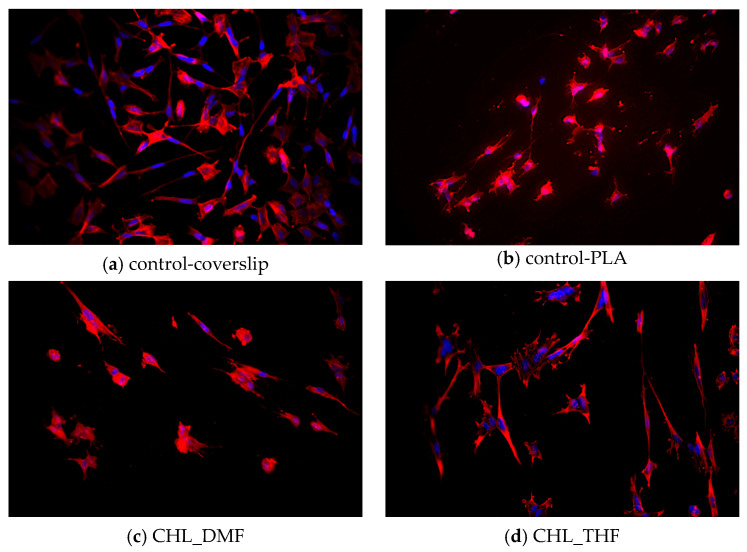

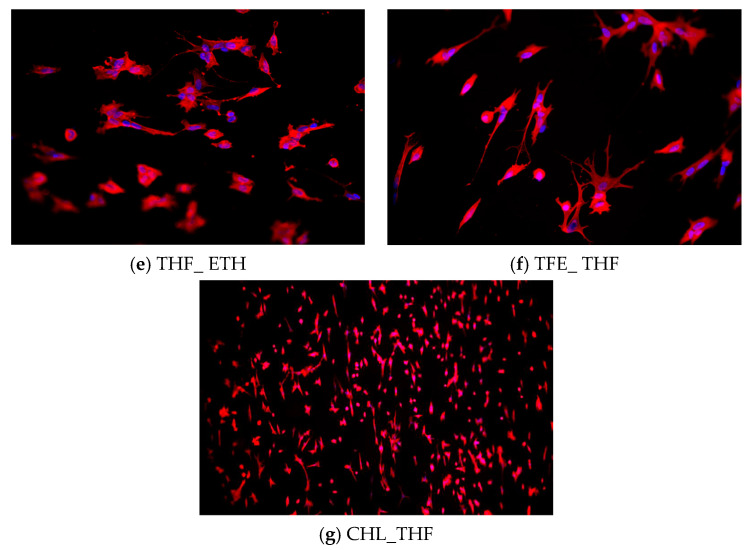
Fluorescence images of SH-SY5Y cells cultured on controls and on scaffolds subjected to VPP at day 12 of differentiation. Cell nuclei are marked in blue, whereas cytoskeletons appear in red.

SH-SY5Y cells were cultured and differentiated for 12 days on the scaffolds of aligned fibers (CHL_DMF, CHL_THF, TFE_ETH, and TFE_THF) subjected to VPP and of control-PLA and on control-coverslips. As shown in Figure 7a, cells cultured on coverslips display a polarized cell body, and some larger neurites randomly extended and established connections to other cells. Cells seeded on scaffolds appear to be at a lower density when compared to coverslip control. This may be due to a lower adhesion to the scaffolds and to a limited observation since, on scaffolds, cells may be at different focusing planes. Regarding morphology, some cells cultured on scaffolds were also able to differentiate from N-type cells, exhibiting neuritic extensions following the alignment pattern of the fibers. Some degree of connection between distinct neuritic extensions is also seen. The control-PLA sample displays manly rounded cells with small neurites. This result is in line with that reported by Londoño et al. [23] found neuronal cells (form NG108-15 cell line) cultured on PLA electrospun scaffold with blebbing plasma membranes, rounded shapes without filopodia or retracting filopodia, characteristic of an unfavorable environment. By contrast, when those cells were cultured on PLA fibers covered by PPy, they showed adequate plasma membranes and neuronal behavior. CHL_THF sample yielded particularly good results, as it apparently presents a higher density of differentiated cells and an increased amount of connecting extensions. The remaining scaffolds seem to have a higher percentage of S-Type cells.

Cell–scaffold interaction depends on multiple physico-chemical and morphological properties of the scaffold. Our results show that the fiber coating by VPP PEDOT may improve the cell–scaffold interaction depending on the solvents used in the preparation of the scaffolds. Among the solvents used to dissolve the oxidant, THF seems to lead to the best results, particularly when this solution is combined with PLA dissolved in CHL, as seen above. It is not possible to correlate cell response to the scaffolds with the conductivity presented in Table 2—as discussed in Section 3.4—that conductivity represents an average over the scaffold apparent volume, while observed cells grew mainly on the surface of the scaffold where conductivity may differ significantly from that average. Beyond conductivity, other surface properties of the scaffolds, such as exposed chemical groups and roughness, may depend on the solvent system used and may influence cell response to the scaffold.

## 4. Conclusions

In this work, fibrous scaffolds were electrospun from PLA solutions containing FeTos and different systems of organic solvents. Fiber preferential alignment was induced in the scaffolds by using a rotatory mandrel as a collector. EDOT polymerization was achieved by VPP, leading to the PEDOT-coating of the fibers. Differences in the chemistry of iron due to the interaction of this ion with different species involved in the scaffolds production impact the polymerization process and the PEDOT properties. Some degree of monomer damage, aside from the polymerization of conductive PEDOT, was confirmed by FTIR spectroscopy. Regardless of the solvent system, all the PEDOT-coated scaffolds displayed the ability to conduct electrical current with estimated conductivities found within the range of the conductivities reported to scaffolds used in neural stimulation studies. However, to study the impact of the electrical stimulation on neural cells making use of the conductive scaffold, the electrical currents associated with stimuli should be measured under the stimulation conditions.

No cytotoxic effects were found for the extracts of PEDOT-coated scaffolds. SH-SY5Y cells were cultured and differentiated on the scaffolds with results that seem to be scaffold dependent concerning the density of differentiated cells and connecting extensions, with the CHL_THF scaffold showing better results.

Overall, the obtained materials display the main requirements needed to proceed with the work in the direction of the in vitro stimulation of neuronal cells.

## Figures and Tables

**Table 1:** Specification of the solutions that were mixed (first and second columns) to achieve the 5 solutions (third column) for electrospinning the scaffolds (fourth column).

PLA Solution (2.5 g)	FeTos Solution: 100 mg FeTos in	Electrospinning Solution Code	Electrospun Scaffold Code
PLA_CHL	100 μL ETH	S1	CHL_ETH
PLA_CHL	530 μL DMF	S2	CHL_DMF
PLA_CHL	225 μL THF	S3	CHL_THF
PLA_TFE	100 μL ETH	S4	TFE_ETH
PLA_TFE	225 μL THF	S5	TFE_THF

**Table 2:** Estimated conductivity for the scaffolds collected at a collector speed of ~3500 rpm.

Scaffold	Conductivity (S/cm)
CHL_DMF	(1.50 ± 0.01) × 10^−1^
CHL_THF	(3.5 ± 0.4) × 10^−7^
TFE_ETH	(7.9 ± 0.2) × 10^−5^
TFE_THF	(1.8 ± 0.1) × 10^−6^

## Data Availability

The data that support the findings of this study are available on request from the corresponding author.
